# Editorial: New GPCR targets and modulators to treat CNS disorders

**DOI:** 10.3389/fnmol.2022.1104336

**Published:** 2022-12-06

**Authors:** Philippe De Deurwaerdère, Evgeni Ponimaskin, Abdeslam Chagraoui, Giuseppe Di Giovanni

**Affiliations:** ^1^Centre National de la Recherche Scientifique, UMR 5287, Bordeaux, France; ^2^Neurophysiology, Hannover Medical School, Hannover, Germany; ^3^Différenciation et Communication Neuroendocrine, Endocrine et Germinale (NorDic), INSERM U1239, IRIB, CHU Rouen, Rouen, France; ^4^Department of Medical Biochemistry, Rouen University Hospital, Rouen, France; ^5^Laboratory of Neurophysiology, Department of Physiology and Biochemistry, Faculty of Medicine and Surgery, University of Malta, Msida, Malta; ^6^Neuroscience Division, School of Biosciences, Cardiff University, Cardiff, United Kingdom

**Keywords:** G-protein coupled receptors, heterodimer, polypharmacology, clinical pharmacology, molecular pharmacology, neuroscience, neurology

G-protein coupled receptors (GPCRs) represent the largest class of human membrane proteins that play critical roles in regulating brain function. GPCRs represent the most important targets in modern pharmacology because of the different functions they mediate, especially within the brain and peripheral nervous system, and also because of their functional and stereochemical properties (Yang et al., 2021). Ligands that selectively activate a single receptor subtype exist for only a small fraction of GPCRs and it has been difficult to develop highly selective ligands for most GPCR subtypes. The molecular approaches have also explored the environment of the GPCR, an essential point to envision that a ligand could trigger distinct responses according to the partners/tissue involved (Lane et al., 2017). In this Research Topic, the GPCRs are considered under the light of the development of radiolabelled probes for *in vivo* studies, the interactome, and integrative response in neuropathic pain or absence epilepsy.

Radioligands have been used for years to study the distribution of GPCR in the CNS. The possibility to follow the binding of radiotracers *in vivo* in humans and small animals was made possible with positron emission tomography using different approaches. Like the *in vitro* binding of radioligands, the *in vivo* binding of radiotracers is conditioned by the state of activity of the GPCR. Colom et al. review the development of ligands for the *in vivo* screening of the distribution of GPCR with a comparison between probes that are agonists or antagonists. The idea is that the binding of agonists would be favored by the active state on the GPCR while the antagonist would bind most states. The review focus on different GPCRs including dopamine D2 receptors, serotonin (5-HT) 1A and 2A receptors, cannabinoid CB1 receptors, muscarinic M2 receptors, histaminergic H3 receptors, μ opioid receptors, κ opioid receptors, and σ1 receptors. They also discuss the pros and cons of using agonists vs. antagonists in using those radiotracers to indirectly study the release of the neurotransmitter (competing with the radiotracers) *in vivo* (Colom et al.).

The state of activity of GPCRs *in vivo* is in part imposed by the protein partners. It has been shown that in addition to the canonical G protein (and β-arrestin) - dependent signaling pathways, GPCR are able to couple to and activate additional signaling molecules, in a G protein- and arrestin-independent manner (Brzostowski and Kimmel, 2001). Sharaf et al. report a proteomic study showing the multiple partners of the CB2 receptor in HEK293 cells. They could determine 83 proteins that could be highly probable interactors with the CB2 receptor. The authors confirmed with one of the possible partners, the scaffold/phagosomal protein p62/SQSTM1, the interaction with CB2 using co-immunoprecipitation and immunocytological studies. The labeling with p62 was associated with vesicular compartments. The authors determined that the ZZ domain of p62 was important for the interaction with the CB2 receptor. They added evidence to the existing literature that the interactome of GPCR comprises complex and functional tangle (Sharaf et al.).

The interactome of GPCR includes intracellular and intramembrane partners as well as proteins or factors of the extracellular matrix. Papon et al. report work on GABA_B_ receptors, a heterodimeric GPCR, at the level of the dorsal horn of the spinal cord. The authors propose that fibulin-2, a protein of the extracellular matrix, inhibits the activity of GABA_B_ receptors by interacting with the B1a subunit of the receptor. Fibulin-2 reduced the inhibitory impact mediated by the stimulation of GABA_B_ receptors in cell culture. In a model of neuropathic pain in rats, fibulin-2 is overexpressed. Its inactivation by molecular strategies like si-RNA *in vivo* augmented the antinociceptive effect induced by the intrathecal administration of the GABA_B_ receptor agonist baclofen. These results highlight the complexity of GABAB receptors (Papon et al.).

It has been previously described that the several GPCR systems [e.g., 5-HT2A/C receptors (Venzi et al., 2016), CB1 receptors (Roebuck et al., 2022)] could be an interesting target in the treatment of epilepsy and comorbid diseases such as anxiety (Crunelli et al., 2020). In a rat model of absence epilepsy, the GAERS rats vs. their non-epileptic controls (NEC), De Deurwaerdère et al. report behavioral and neurochemical effects of the preferential CB_1/2_ receptor agonist WIN 55,212-2. Using quantitative and Temporal pattern (T-pattern) analyses, they reported that the agonist was anxiolytic in NEC rats but was sedative in GAERS rats. The balanced behavioral effects of WIN 55,212-2 between GAERS and NEC were associated with distinct levels of monoamine tissue content mainly in the output regions of the basal ganglia (substantia nigra and entopeduncular nucleus) and hippocampus out of the 14 brain regions analyzed. The epileptic status is possibly associated with changes of endocannabinoid system and hypersensitive responses to CB receptors converging to the basal ganglia. At least, as far as monoamines are concerned, the effects of WIN 55,212-2 are regionally restricted and maybe due to local and specific actions of CB1 receptors (De Deurwaerdère et al.).

In conclusion, the new tools have the promise to provide unprecedented insights into the biology and circuitry underlying numerous CNS diseases. It might start with pertinent tools to address the state of activity of GPCRs *in vivo*. The interactome is likely cell dependent and additional data could go in that way in order to understand regional responses to drugs. These discoveries will inform drug discovery efforts on how to optimally steer receptor signaling in a given patient population to provide maximal efficacy with minimal side effects and provide exciting opportunities for the treatment of CNS orders.

## Author contributions

All authors listed have made a substantial, direct, and intellectual contribution to the work and approved it for publication.
